# Real-World Clinical Experience of Rosuvastatin as a Lipid-Lowering Therapy for Primary and Secondary Prevention of Cardiovascular Events (REAL ROSE)

**DOI:** 10.7759/cureus.31468

**Published:** 2022-11-14

**Authors:** Ashwani Mehta, Pradeep Jain, Ravikant Patil, T Sashi Kant, Sanjiv A Indurkar, Sunil Kumar Kota, Santosh Revankar, Amit Gupta

**Affiliations:** 1 Cardiology, Dr Ashwani Mehta Clinic, New Delhi, IND; 2 Cardiology, Indraprastha Apollo Hospitals, New Delhi, IND; 3 Internal Medicine, Sevasadan Lifeline Superspecialty Hospital, Miraj, IND; 4 Internal Medicine, Sri Sai Heart Care Clinic, Hyderabad, Hyderabad, IND; 5 Internal Medicine, Diabetes Clinic, Aurangabad, IND; 6 Internal Medicine, Diabetes & Endocare Clinic, Berhampur, IND; 7 Scientific Services, USV Private Limited, Mumbai, IND

**Keywords:** rosuvastatin, statins, high-intensity, dyslipidemia, dose titration

## Abstract

Background

Rosuvastatin effectively reduces endogenous cholesterol synthesis and low-density lipoprotein (LDL) cholesterol and increases high-density lipoprotein (HDL) cholesterol. This study aimed to evaluate the clinical characteristics of patients and treatment patterns of rosuvastatin as a lipid-lowering therapy for primary and secondary prevention of cardiovascular events in Indian settings.

Methods

This real-world, retrospective multi-centric observational study included patients aged >18 years who received treatment with a rosuvastatin/rosuvastatin-based combination. Demographic and data about concomitant diseases and medications were recorded.

Results

Out of 1,816 patients, the majority were men (66.2%); the mean age was 54.1 years. The patients prescribed rosuvastatin for primary and secondary prevention of cardiovascular events were 71.9% and 28.1%, respectively. Rosuvastatin 10 mg (56.8%) was the most commonly prescribed dose. For primary prevention, 10 mg (65.0%) was the most preferred dose, and for secondary prevention, 20 mg (54.3%) was the most preferred dose. Rosuvastatin treatment significantly (pre- vs. post-treatment) reduced the levels of total cholesterol (227.2 vs. 178.4 mg/dL), triglycerides (212.6 vs. 154.4 mg/dL), and LDL cholesterol (167.0 vs. 125.6 mg/dL), and increased HDL cholesterol levels (40.7 vs. 44.3 mg/dL) (p<0.0001). A total of 1,196 patients received combination therapy with rosuvastatin (aspirin, 34.0%, and fenofibrate, 21.9%). Adverse events were reported in 0.4% of the study population (leg pain, nausea, muscle cramps/pain, bleeding, and myalgia).

Conclusion

This study demonstrated the clinical effectiveness and safety of moderate- to high-intensity rosuvastatin (5-40 mg) for primary and secondary prevention of cardiovascular events in the Indian population. A primary prevention strategy with statins can reduce cardiovascular events and associated morbidity and mortality.

## Introduction

The rapidly increasing prevalence of cardiovascular diseases (CVDs) and the CVD-associated risk factors among Indians have substantially contributed to the increased mortality due to CVDs. Currently, CVDs contribute to almost 25% of deaths in India, and the majority (>80%) of this burden is attributed to ischemic heart disease and stroke [1,2]. In addition to the CVDs that occur primarily, the development of secondary cardiovascular events in patients with past CVD or CVD-associated risk factors has posed a major challenge in the reduction of premature CVD deaths. It is of utmost importance to prevent not only secondary cardiovascular events but also primary cardiovascular events.

Among several risk factors associated with the development of CVDs, dyslipidemia is one of the crucial modifiable risk factors that can be used as a disease-modifying target for the management of CVDs [3,4]. Evidence across the world has supported the role of maintenance of lipid levels within the normal range in reducing the morbidity and mortality associated with adverse cardiovascular events [5]. The lipid-lowering therapy benefits primary as well as secondary prevention of cardiovascular events.

Statins are the cornerstone of anti-lipid therapy or dyslipidemia management. Across the wide range of available statins, rosuvastatin is effective in reducing the endogenous cholesterol synthesis through inhibition of the 3-hydroxy-3-methylglutaryl coenzyme A (HMG-CoA) reductase enzyme. In the cholesterol biosynthetic pathway, this enzyme converts HMG-CoA into mevalonic acid, which is the rate-limiting step [6]. Along with reducing total cholesterol, rosuvastatin is also effective in reducing low-density lipoprotein (LDL) cholesterol in the blood and elevates the high-density lipoprotein (HDL) cholesterol levels by 15%. No other statin has shown the benefit of increasing HDL cholesterol to this extent [5-7]. Various clinical studies have reported better efficacy of rosuvastatin in reducing LDL cholesterol, increasing HDL cholesterol, and being useful in achieving lipid goals in a greater number of patients than other statins. It is also vital in the regression of coronary atherosclerosis and has a potential role in patients for secondary prevention [7]. Furthermore, the availability of rosuvastatin in a wide range of doses ranging from 5 mg to 40 mg gives physicians an adjustment window for treatment doses to achieve maximum benefit as per the requirement of the individual patients, and, at the same time, it also aids to minimize the side effects [5,8]. According to the European Society of Cardiology (ESC) and European Atherosclerosis Society (EAS) 2019 guidelines, response to statin treatment varies individually, and it is recommended to consider up-titration of the statin dose before initiating additional LDL-lowering treatments for the lipid modification to reduce cardiovascular risk [9]. In the American Diabetes Association (ADA) 2020 guidelines, the use of moderate-intensity statins is recommended for primary prevention in patients with diabetes aged 20-39 years with additional atherosclerotic cardiovascular disease (ASCVD) risk factors and in patients aged 40-75 years without ASCVD risk factors, while the use of high-intensity statins is indicated for secondary prevention in patients of all ages with diabetes and atherosclerotic CVD [10].

The present real-world observational study retrospectively analyzed the clinical characteristics of patients and treatment patterns including different dosage forms of rosuvastatin or rosuvastatin-based combination as a lipid-lowering therapy for primary and secondary prevention of cardiovascular events in the Indian settings.

## Materials and methods

Study design

This was a real-world, retrospective non-randomized, non-comparative, multi-centric observational study conducted across 73 Indian healthcare centers having medical records of adult patients who had received treatment with rosuvastatin or rosuvastatin-based combination as a lipid-lowering therapy for primary and secondary prevention of cardiovascular event.

Ethics

This study was conducted in accordance with the ethical principles that are consistent with the Declaration of Helsinki, the International Conference on Harmonization Good Clinical Practices, and the applicable legislation on non-interventional studies. The study protocol was approved by Independent Ethics Committee (ACEAS - Independent Ethics Committee, Ahmedabad, Gujarat, India) prior to the commencement of the study.

Inclusion and exclusion criteria

Retrospectively identified patients of either sex, aged 18 years or older, who have received treatment for dyslipidemia with rosuvastatin or rosuvastatin-based combination, and whose treating physician agreed to provide information regarding their treatment were included. Patients having incomplete data were excluded from the study.

Data collection

Data related to demographic characteristics, duration of disease, comorbidities, concomitant medications, and rosuvastatin dosage patterns were collected from patients’ medical records available at the hospital/clinics and authenticated by physicians during routine care.

Endpoints

The aim was to determine patterns of different dosages of rosuvastatin (5/10/20/40 mg) and rosuvastatin-based combination in primary and secondary prevention, post-therapy change in lipid profile parameters with different dosages of rosuvastatin, the pattern of rosuvastatin dose modification required during the course of treatment, the spectrum of comorbid conditions, and adverse events reported in the last one year related to statin therapy.

Statistical analysis

Data were analyzed using the Statistical Package for the Social Sciences (SPSS) Version 23.0 (IBM Corp., Armonk, NY, USA). Continuous variables were summarized with descriptive statistics, including mean and standard deviation (SD), and categorical variables were presented as frequency and percentages. A comparison of qualitative and quantitative variables between the groups was done using the chi-square test and Mann-Whitney U test, respectively. A paired sample t-test was used for comparing the pre- and post-treatment lipid levels. A p-value of <0.05 was considered statistically significant.

## Results

A total of 1,816 patients (66.2% male and 33.8% female) were enrolled in this study. The mean age and body mass index of the overall population was 54.1 years and 27.3 kg/m2, respectively. In the present study, 61.9% of the study population belonged to the age group of ≥40 to ≤60 years. The majority of patients were from urban and semi-urban areas (78.5%). Among the risk factors, excess salt intake (74.2%) was the most commonly observed risk factor, followed by stress (48.2%), smoking (44.3%), and family history of coronary artery disease (CAD) (21.7%) (Table 1).

**Table 1:** Baseline demographics and treatment details Data are presented as n (%) unless otherwise specified. BMI, body mass index; CAD, coronary artery disease; DAPT, dual antiplatelet therapy; SD, standard deviation

Parameters	Number of patients (N=1,816)
Age (years), mean (SD)	54.1 (10.9)
Weight (kg), mean (SD)	72.7 (10.9)
Height (cm), mean (SD)	163.4 (8.5)
BMI (kg/m^2^), mean (SD)	27.3 (4.0)
Treatment duration (months), mean (SD)	16.5 (18.6)
Gender	
Men	1202 (66.2)
Women	614 (33.8)
Occupation (n=1158)	
Employed	489 (42.2)
Self-employed	340 (29.4)
Homemaker	258 (22.3)
Labor	7 (0.6)
Retired	56 (4.8)
Unemployed/student	8 (0.7)
Location (n=1,664)	
Rural	318 (19.1)
Semi-rural	40 (2.4)
Semi-urban	364 (21.9)
Urban	942 (56.6)
Excess salt intake	1348 (74.2)
Smoking	805 (44.3)
Coping with stress	876 (48.2)
Family history of CAD	394 (21.7)
Rosuvastatin dose	
5 mg	205 (11.3)
10 mg	1032 (56.8)
20 mg	530 (29.2)
40 mg	49 (2.7)
Dose titration (n=1794)	
Up-titration	487 (27.1)
Down-titration	155 (8.6)
No titration	1152 (64.2)
Combination therapy with rosuvastatin	1196 (65.9)
Aspirin	618 (34.0)
Fenofibrate	398 (21.9)
Clopidogrel	226 (12.4)
Vitamin D	206 (11.3)
Coenzyme Q10	92 (5.1)
DAPT	84 (4.6)
Omega 3 fatty acids	59 (3.2)
Saroglitazar	12 (0.7)

The proportion of patients prescribed rosuvastatin for primary and secondary prevention of cardiovascular events was 71.9% (n=1305) and 28.1% (n=511), respectively (Tables 2, 3). In the overall study population, the most commonly used dose of rosuvastatin was 10 mg (56.8%), followed by 20 mg (29.2%), 5 mg (11.3%), and 40 mg (2.7%) of patients (Table 1).

**Table 2:** Analysis of treatments based on medical history (primary prevention) Data are presented as n (%) unless otherwise specified. DAPT, dual antiplatelet therapy; DM, diabetes mellitus

Parameters	Rosuvastatin				Total
	5 mg (n=205)	10 mg (n=1032)	20 mg (n=530)	40 mg (n=49)	N=1,816
Primary prevention	153 (11.7)	849 (65.0)	293 (22.5)	10 (0.8)	1305 (71.9)
Medical history					
Dyslipidemia	87 (9.2)	651 (67.9)	211 (22.0)	9 (0.9)	958 (73.4)
Type 2 DM	90 (13.0)	452 (65.4)	149 (21.5)	1 (0.1)	692 (53.0)
Hypertension	63 (8.8)	460 (63.9)	193 (26.9)	3 (0.4)	719 (55.1)
Concomitant medications					
Aspirin	25	210	134	6	375 (28.7)
Fenofibrate	11	210	51	0	272 (20.8)
Vitamin D	17	98	26	2	143 (11.0)
Clopidogrel	6	38	55	3	102 (7.8)
Coenzyme Q	6	14	45	0	65 (5.0)
Omega 3 fatty acid	0	34	8	1	43 (3.3)
DAPT	0	8	6	0	14 (1.1)
Saroglitazar	0	6	1	0	7 (0.5)

**Table 3:** Analysis of treatments based on medical history (secondary prevention) Data are presented as n (%) unless otherwise specified. ACS, acute coronary syndrome; CAD, coronary artery disease; DAPT, dual antiplatelet therapy; PAD, peripheral artery disease

Parameters	Rosuvastatin				Total
	5 mg (n=205)	10 mg (n=1032)	20 mg (n=530)	40 mg (n=49)	N=1816
Secondary prevention	25 (4.9)	165 (32.4)	278 (54.3)	43 (8.4)	511 (28.1)
Medical history					
ACS	1 (1.3)	9 (11.3)	56 (70.9)	13 (16.5)	79 (15.5)
Stroke	-	19 (51.4)	14 (37.8)	4 (10.8)	37 (7.2)
Stable CAD	5 (2.8)	46 (26.1)	92 (52.3)	13 (7.3)	176 (34.4)
PAD	1 (11.1)	2 (22.2)	6 (66.7)	-	9 (1.8)
Heart failure	-	3 (37.5)	3 (37.5)	2 (25.0)	8 (1.6)
Concomitant medications					
Aspirin	6	67	136	31	240 (46.9)
Fenofibrate	4	25	65	13	107 (20.9)
Clopidogrel	1	25	63	10	99 (19.4)
DAPT	5	7	53	1	66 (12.9)
Vitamin D	3	6	35	7	51 (10.0)
Omega 3 fatty acid	0	1	14	1	16 (3.1)
Coenzyme Q	0	0	8	1	9 (1.8)
Saroglitazar	1	0	3	1	5 (1.0)

The mean duration of treatment was significantly higher in patients receiving 20 mg of rosuvastatin (20.0 months) when compared to patients receiving 5 mg (14.3 months), 10 mg (15.1 months), and 40 mg (16.8 months) rosuvastatin (p<0.0001).

Primary prevention

For primary prevention of cardiovascular events, moderate-intensity rosuvastatin therapy was the most commonly used therapy (10 mg, 65.0%) in the overall population. In patients with a history of dyslipidemia (n=958), 10 mg of rosuvastatin was used in 67.9% of the population followed by 20 mg dose in 22.0% of patients. Similar trend was also observed in patients with diabetes (n=692) and hypertension (n=719), where 10 mg and 20 mg of rosuvastatin were used in 65.4% and 63.9% and 21.5% and 26.9% of the patient population, respectively (Table 2).

Secondary prevention

For secondary prevention of cardiovascular events, high-intensity rosuvastatin therapy was used in majority of the population (20 mg, 54.3%). In post-acute coronary syndrome (ACS) patients, the majority were on high-intensity statin, of which 70.9% received 20 mg and 16.5% received 40 mg of rosuvastatin. In patients with a history of stroke, 10 mg of rosuvastatin was used in majority of patients (51.4%) followed by 20 mg (37.8%) and 40 mg (10.8%) of dosages. In patients with a history of stable CAD, 20 mg (52.3%) and 10 mg (26.1%) doses of rosuvastatin were most commonly used. In patients with a history of heart failure and peripheral arterial disease (PAD), majority of patients were on high-intensity statin therapy (heart failure: 20 mg, 37.5% and 40 mg, 25.0%; PAD: 20 mg, 66.7%) followed by moderate-intensity statin therapy (heart failure: 10 mg, 37.5%, and PAD: 10 mg, 22.2%, respectively) (Table 3).

Effect of rosuvastatin on lipid parameters

**Figure 1:**
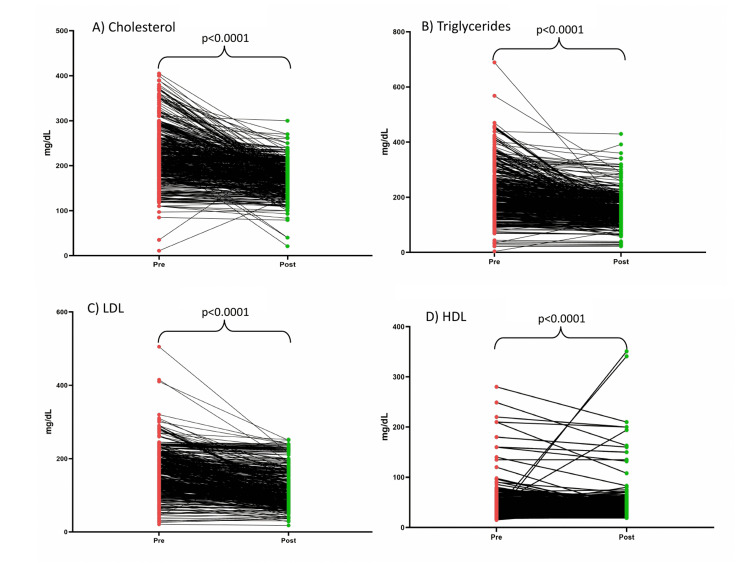
Lipid parameters before and after treatment with rosuvastatin. LDL; low-density lipoprotein; HDL, high-density lipoprotein

A significant reduction in total cholesterol, LDL cholesterol, and triglycerides levels was observed post-rosuvastatin treatment across all the four doses (5, 10, 20, and 40 mg) (p<0.05). Rosuvastatin at 10 mg (40.3 vs 42.8 mg/dL; p<0.0001) and 20 mg (40.4 vs. 44.4 mg/dL; p=0.044) doses were effective in significantly increasing HDL cholesterol levels from pre-treatment. In the overall population, post-treatment rosuvastatin significantly reduced the levels of total cholesterol (227.2 vs. 178.4 mg/dL; p<0.0001), triglycerides (212.6 vs. 154.4 mg/dL; p<0.0001), and LDL cholesterol (167.0 vs. 125.6 mg/dL; p<0.0001), and significantly increased the levels of HDL cholesterol (40.7 vs. 44.3; p<0.0001) compared to pre-treatment levels (Figure 1).

Combination therapy with rosuvastatin

In the overall population, among 1,196 (65.9%) patients receiving combination therapy with rosuvastatin, aspirin (34.0%) and fenofibrate (21.9%) were the most frequently used combination drugs (Table 1).

Rosuvastatin with aspirin combination was used in 28.7% (n=375) of the primary prevention cohort (N=1305), where majority of patients were receiving 10 mg of rosuvastatin (n= 210) followed by 20 mg (n=134) (Table 2). Among patients receiving combination therapy in the secondary prevention group (N=511), 46.9% (n=240) of patients were on rosuvastatin/aspirin combination, whereas 20.9%, 19.4%, and 12.9% of patients received fenofibrate, clopidogrel, and dual antiplatelet therapy (DAPT), respectively, as a combination drug with rosuvastatin. For secondary prevention, combination therapy was most commonly used with 20 mg of rosuvastatin (aspirin, fenofibrate, clopidogrel, DAPT, vitamin D, omega 3 fatty acid, coenzyme Q, and saroglitazar). In a small proportion of patients, rosuvastatin was also used as a combination therapy along with vitamin D, omega 3 fatty acid, coenzyme Q, and saroglitazar (Table 3).

Age-wise analysis

Dyslipidemia was the most prevalent condition across all the age groups (80.4%-81.9%). Hypertension and type 2 diabetes mellitus were more common in elderly age group (>60 years) (68.7% and 62.4%) followed by patients in the age group of ≥40 to ≤60 (62.7% and 58.5%) and >18 to <40 years (50.8% and 45.2%) (p<0.0001). Proportion of patients with a medical history of ACS or stable CAD were more common in the elderly age group than other age groups (p=0.002 and p<0.0001, respectively). A significantly decreasing trend was observed in the use of rosuvastatin for primary prevention of cardiovascular events from the youngest to oldest age groups (>18 to <40 years [84.4%]; ≥40 to ≤60 years [74.3%]; >60 years [61.1%]) (p<0.0001). However, a significantly increasing trend was observed in the use of rosuvastatin for secondary prevention of cardiovascular events from the oldest to youngest age groups (>18 to <40 years [15.6%]; ≥40 to ≤60 years [25.7%]; >60 years [38.9%]) (p<0.0001) (Table 4).

**Table 4:** Age-wise analysis Data are presented as n (%) unless otherwise specified. ^a^n=160. ^b^n=958. ^c^n=417. ACS, acute coronary syndrome; BMI, body mass index; CAD, coronary artery disease; DM, diabetes mellitus; PAD, peripheral artery disease; SD, standard deviation; TIA, transient ischemic attack

Parameters	Age group (years)			p-Value
	>18 to <40 (n=199)	≥40 to ≤60 (n=1125)	>60 (n=492)	
BMI (kg/m^2^), mean (SD)	26.7 (4.0)^a^	27.5 (4.0)^b^	27.2 (3.7)^c^	0.069
Salt intake	139 (69.8)	855 (76.0)	354 (72.0)	0.075
Smoking	76 (38.2)	486 (43.2)	243 (49.4)	0.013
Stress	100 (50.3)	580 (51.6)	196 (39.8)	<0.0001
Family history of CAD	61 (30.7)	228 (20.3)	105 (21.3)	0.005
Medical history				
Dyslipidemia	163 (81.9)	904 (80.4)	400 (81.3)	0.827
Type 2 DM	90 (45.2)	658 (58.5)	307 (62.4)	<0.001
Hypertension	101 (50.8)	705 (62.7)	338 (68.7)	<0.001
Stable CAD	19 (11.4)	110 (9.5)	89 (18.1)	<0.001
ACS	18 (9.0)	70 (6.2)	55 (11.2)	0.002
Heart failure	1 (0.5)	6 (0.5)	8 (1.6)	0.072
Stroke/TIA	4 (2.0)	24 (2.1)	15 (3.0)	0.505
PAD	1 (0.5)	6 (0.5)	5 (1.0)	0.522
Other	5 (2.5)	21 (1.9)	2 (0.4)	0.045
Primary prevention	168 (84.4)	836 (74.3)	301 (61.1)	<0.0001
Secondary prevention	31 (15.6)	289 (25.7)	191 (38.9)	<0.0001

Dose titration pattern

During the course of treatment, rosuvastatin dose titration was not done in 64.2% patient population, while up-titration was done in 27.1% and down-titration in 8.6% of the overall patient population (Table 1). Among patients who underwent up-titration of rosuvastatin, uncontrolled lipid level was the most common reason for up-titration of rosuvastatin (44.6%). Presence of multiple risk factors such as sedentary lifestyle, smoking, and stress was also considered as a reason for up-titration of rosuvastatin dosages in the remaining population. In patients who underwent down-titration of rosuvastatin, controlled lipid levels (48.4 %) appeared as the most common reason for down-titration. High dose intolerance was also mentioned as a reason for down-titration in 1.3% of patients (Table 5).

**Table 5:** Reasons for titration in patients receiving rosuvastatin

Reasons for titration	Number of patients (%)
Up-titration (n=487)	
Uncontrolled lipid levels	217 (44.6)
Stress and sedentary lifestyle	129 (26.5)
Smoking	65 (13.3)
Reason not reported	42 (8.6)
Others	34 (7.0)
Down-titration (n=155)	
Controlled lipid levels	75 (48.4)
Reason not reported	55 (35.5)
High dose intolerance	2 (1.3)
Others	23 (14.8)

Adverse events were reported in a total of eight patients, which include leg pain (n=3), nausea (n=2), and muscle cramps/pain, bleeding, and myalgia (n=1, each).

## Discussion

This real-world study assessed the clinical experience of the use of rosuvastatin as a lipid-lowering therapy for primary and secondary prevention of cardiovascular events in Indian settings. The majority of the present study population was from the age group ≥40 to ≤60 years and male patients were prevalent. Overall observations of the present study indicate that doses of rosuvastatin raging between 5 and 20 mg were preferred for primary prevention and 20 and 10 mg dosages of rosuvastatin were preferred for secondary prevention of cardiovascular events by physicians from India.

In patients with a history of type 2 diabetes mellitus, dyslipidemia, or hypertension, 10 mg of rosuvastatin was the predominant therapy for primary prevention. These observations are in concordance with the ADA 2020 guidelines, wherein moderate-intensity statin therapy is recommended for primary prevention in diabetes patients aged 40 to 75 years without ASCVD risk factors and younger patients aged 20 to 39 years with ASCVD risk factors. The ADA 2020 guidelines also recommend high-intensity statin therapy for primary prevention in diabetes patients at higher risk, especially those with multiple ASCVD risk factors [10]. In the present study, 21.5% of patients with a history of type 2 diabetes mellitus were on high-intensity rosuvastatin (20 mg) therapy. According to the Lipid Association of India guidelines on the management of dyslipidemia, the use of statins is considered as a safe and effective strategy for primary prevention of ASCVD and should be guided by the estimated ASCVD risk in a given individual. It is indicated to use high‑intensity rosuvastatin (20-40 mg/day) therapy to achieve >50% LDL cholesterol reduction from baseline and moderate‑intensity rosuvastatin (5-10 mg/day) therapy to achieve 30 to <50% LDL cholesterol reduction from baseline [8]. There may be patients in whom 40 mg rosuvastatin may be initiated initially, and once the lipid levels are under control, patients may be shifted to 20 mg as maintenance therapy.

In the present study, various rosuvastatin-based combination therapies were used for the management of primary or secondary prevention of cardiovascular events. Among these, rosuvastatin with aspirin combination was the most commonly used therapy for the primary prevention cohort, and 10 mg of rosuvastatin followed by 20 mg of rosuvastatin were the most common doses used in combination with aspirin.

Many RCTs and meta-analyses have confirmed that routine early use of high-intensity statin therapy is associated with rapid and long-term clinical benefits in patients with ASCVD [11-13]. In the present study, majority of post-ACS patients were on high-intensity statin, of which 70.9% were on 20 mg of rosuvastatin. However, to achieve the LDL cholesterol goal of <55 mg/dL as recommended by the recent ESC 2019 guideline, there is a scope for further intensification of statin therapy to the maximum tolerated statin dose, and the addition of ezetimibe to statin therapy can be considered in these patients [9]. A previous Indian survey that provided useful insights into Indian physicians’ self-reported perspectives on managing dyslipidemia in routine clinical practice reported usage of high-intensity statins in post-ACS cases (atorvastatin at 40/80 mg or rosuvastatin at 20/40 mg [73.7%]) [14].

Secondary prevention is very important in patients who had a stroke and transient ischemic attack (TIA) in the past as there is an increased risk of recurrent cerebrovascular events, as well as other major cardiovascular events including myocardial infarction (MI) post-treatment. Statins therapy, one of the established secondary prevention therapies, reduces the risk of recurrent stroke (by 12% per mmol/L reduction in LDL cholesterol), MI, and vascular death [15,16]. More than 50% of patients with a history of stroke in the present study were on moderate-intensity statin therapy (10 mg of rosuvastatin), while around 47% of patients were on high-intensity statin therapy. The 2019 update of the American Heart Association (AHA) 2018 stroke guideline and the ESC 2019 guideline recommend the use of high-intensity statin therapy in patients with a history of TIA or ischemic stroke [9-17]. In the Lipid Association of India guidelines, statin treatment is indicated for patients with acute ischemic stroke, for primary prevention of stroke in adults with diabetes or CAD, and for secondary prevention of stroke as well [18].

The burden of dyslipidemia is increasing in the Indian population, with a recent report from the Indian Council of Medical Research-India Diabetes (ICMR-INDIAB) study indicating higher rates of dyslipidemia in the youngest age group (20-24 years) [3,4]. Another real-world study from India also provided corroborating evidence that included young adult patients with dyslipidemia and reported the highest prevalence of atherogenic dyslipidemia among the youngest individuals (≥18 to ≤25 years) than individuals from the 25-45 years of age group [19]. However, in patients with LDL cholesterol < 190 mg/dL, an individual’s 10-year ASCVD risk needs to be considered to deciding on statin therapy [20]. The Indian study that evaluated the use of glimepiride and metformin combination to control type 2 diabetes mellitus in Indian patients reported concomitant use of statin to manage dyslipidemia [21].

PAD is a typical characteristic of atherosclerosis, and patients with PAD are at very high risk of coronary events and subsequent mortality due to CVD. Therefore, it is crucial to treat these patients with a therapeutic regimen that achieves ≥50% LDL cholesterol reduction from baseline [9]. In the present study, a total of 66.7% of patients with PAD were on 20 mg of rosuvastatin and 22.2% were on 10 mg of rosuvastatin. According to the ESC 2019 guidelines, patients with PAD should be treated with a lipid-lowering therapy with a maximum tolerated dose of statin plus ezetimibe or a combination with a PCSK9 inhibitor (if needed) to reduce the risk of ASCVD events.

Reducing the risk of heart failure with lipid-lowering statin therapy in patients with CAD is a well-established fact, and several international treatment guidelines recommend statin therapy for secondary prevention in patients with CAD [22-24]. In this study, around 60% of patients with stable CAD were on high-intensity rosuvastatin therapy.

In the majority of the present study population, rosuvastatin dose titration was not done during the treatment course. Among the patients who required dose titration, uncontrolled lipid levels appeared as the major reason for dose intensification. Other risk factors that attributed to the intensification of rosuvastatin were sedentary lifestyle, stress, and smoking. These observations suggest the importance of close monitoring of lipid levels in the patients taking rosuvastatin during treatment.

In the present study, all four doses of rosuvastatin were effective in reducing the total cholesterol, triglycerides, and LDL cholesterol levels. These results showed the effectiveness of a low dose of rosuvastatin in reducing LDL cholesterol levels, suggesting rosuvastatin as the most potent statin in cardiovascular risk reduction.

Aspirin administration at low doses helps prevent ASCVD and has been in routine clinical practice for decades. It is a widely accepted treatment for secondary prevention of cardiovascular events [25-28]. The 2019 American College of Cardiology/American Heart Association (ACC/AHA) guideline on the primary prevention of CVD recommends aspirin at low doses (75-100 mg) in patients aged 40-70 years who are at a high risk of ASCVD [29]. According to the ADA 2020 guidelines, aspirin therapy (75-162 mg/day) can be used for primary prevention in patients with diabetes and a high risk of CVD; however, patients should be involved in decision-making based on the benefits/risk ratio [10]. In the present study, rosuvastatin with aspirin combination was used in around 29% of the primary prevention cohort and 47% of the secondary prevention cohort.

Adverse events were reported in 0.4% of study populations, a very small part of the population, suggesting that all four doses of rosuvastatin were well tolerated in the present study population.

This study is limited by its retrospective and observational nature, and more studies may be needed to support our results. Additionally, the lack of real-world evidence with respect to clinical experience of rosuvastatin treatment in primary and secondary prevention has restricted the comparison of the present study observations with any other real-world study. Therefore, more real-world studies on rosuvastatin will be beneficial for a deeper understanding of treatment patterns used in routine clinical practice.

## Conclusions

This retrospective real-world study demonstrated the clinical effectiveness and safety of moderate- to high-intensity rosuvastatin (5-40 mg) for primary and secondary prevention of cardiovascular events in the Indian population. Indian physicians preferred rosuvastatin 10 mg for primary prevention and high-intensity rosuvastatin (20 mg) for secondary prevention. Considering the burden of hypertension and diabetes population in India, a primary prevention strategy with statins can reduce cardiovascular events and associated morbidity and mortality.
